# Novel approaches to modulate CAR-T cell function by targeting the tumor microenvironment in ovarian cancer

**DOI:** 10.1186/s13048-026-02149-z

**Published:** 2026-06-19

**Authors:** Saber SamadiAfshar, Hossein Azizi, Meghdad Yeganeh, Sahel SamadiAfshar, Thomas Skutella

**Affiliations:** 1 Pediatric Health Research Center, Tabriz University of Medical Sciences, Tabriz, 5143377505 Iran; 2 Department of Stem Cells and Cancer, College of Biotechnology, Amol University of Special Modern Technologies, Amol, 49767 Iran; 3 School of Biotechnology, College of Science, University of Tehran, Tehran, 1417935840 Iran; 4 Research Development Unit, Taleghani Hospital, Tabriz University of Medical Sciences, Tabriz, 5143377505 Iran; 5 Institute for Anatomy and Cell Biology, Medical Faculty, University of Heidelberg, Im Neuenheimer Feld 307, 69120 Heidelberg, Germany

**Keywords:** Ovarian Cancer, CAR-T cell therapy, Tumor microenvironment, Immunotherapy, Precision oncology

## Abstract

Ovarian cancer (OC) remains one of the most lethal gynecological malignancies despite advances in conventional therapeutic approaches. The dismal approximately 30% five-year survival rate for advanced disease highlight the urgent need for innovative treatment strategies. Chimeric antigen receptor T (CAR-T) cell therapy, having revolutionized treatment paradigms in hematological malignancies, faces significant challenges when applied to the complex immunosuppressive tumor microenvironment (TME) of ovarian cancer. This comprehensive review investigates critical research questions regarding the extent to which cellular and molecular components of the ovarian cancer TME inhibit CAR-T cell cytotoxic function, and the signaling patterns associated with reduced CAR-T cell infiltration or persistence in tumor masses. We systematically examine the multifaceted immunosuppressive mechanisms within the ovarian cancer TME and evaluate breakthrough strategies designed to overcome these barriers, including next-generation CAR engineering, combinatorial therapeutic approaches, and innovative TME-modulating technologies. The insights presented here provide a crucial roadmap for translating CAR-T therapy from promising concept to clinical reality in ovarian cancer treatment, potentially transforming outcomes for patients with this recalcitrant malignancy where innovative therapeutic options are urgently needed.

**Graphical Abstract:**

**Figure:**
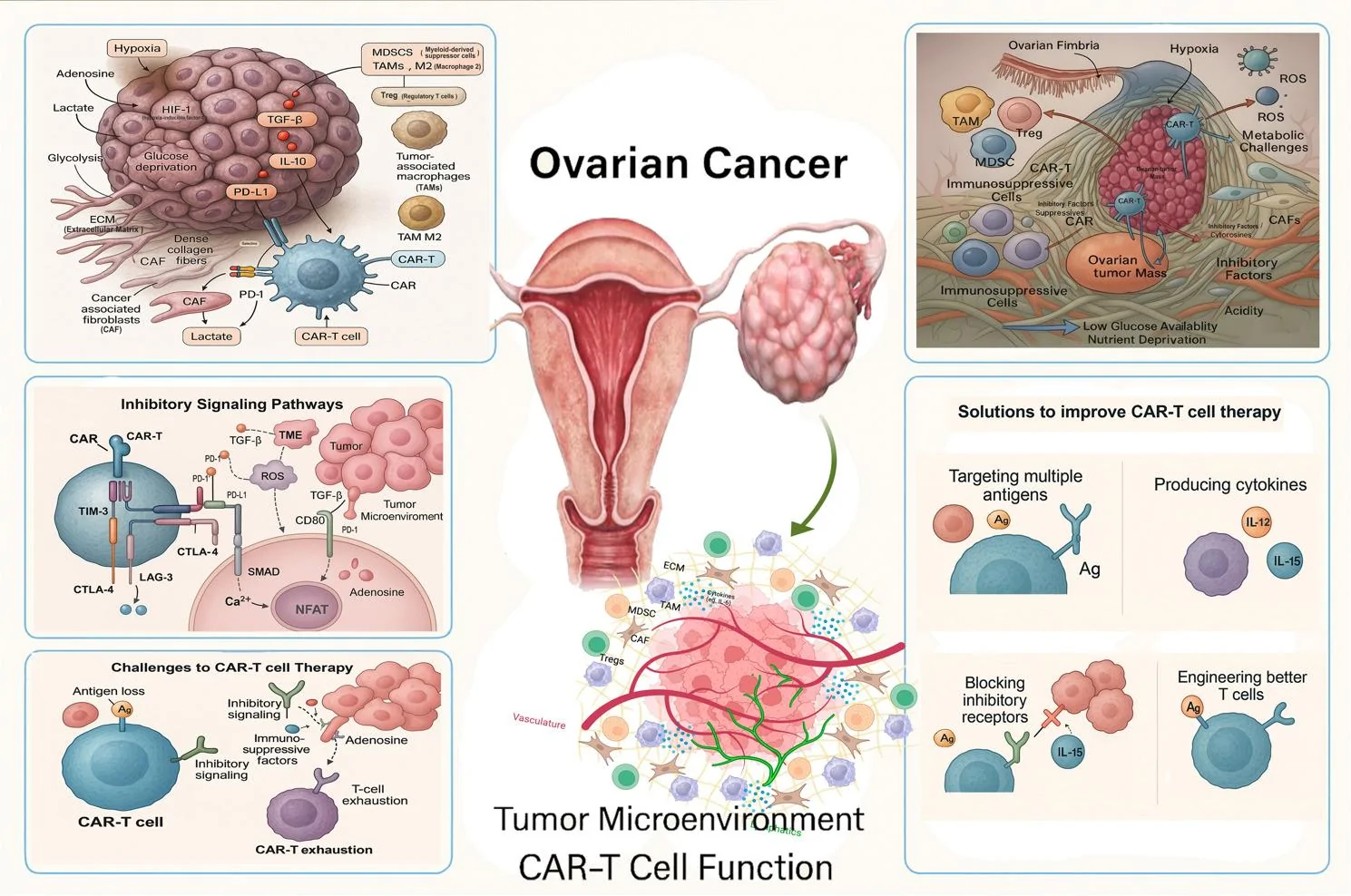

## Highlights

Novel CAR-T strategies enhance efficacy in the immunosuppressive ovarian TME.

Hypoxia and metabolic stress limit CAR-T cell persistence and cytotoxic function.

Armored CAR-T cells resist TGF-β signaling and improve intratumoral activity.

CRISPR editing enables checkpoint-resistant and durable CAR-T cell responses.

Nanoparticles deliver modulators that reprogram TME to support CAR-T therapy.

Engineered probiotics modulate immune tone and support CAR-T infiltration.

Metabolic conditioning enhances CAR-T function in nutrient-deprived tumors.

Multi-antigen CAR-Ts reduce escape and increase targeting precision in EOC.

Translational hurdles include scalability, regulatory gaps, and treatment costs.

Integrative, personalized TME-targeting may unlock CAR-T potential in EOC.

## Introduction

Ovarian cancer (OC) remains one of the most lethal gynecological malignancies globally, with approximately 313,000 new cases and 207,000 deaths reported annually according to recent global cancer statistics [1]. Despite significant advances in conventional therapeutic approaches—including cytoreductive surgery, platinum-based chemotherapy regimens, radiation therapy, and targeted therapies such as PARP inhibitors—the five-year survival rate for patients with advanced-stage disease remains discouraging at approximately 30% [2, 3]. This poor prognosis primarily stems from late-stage diagnosis, high recurrence rates, and the development of therapeutic resistance, underscoring the critical need for innovative treatment strategies [4].

In the evolving landscape of cancer therapeutics, CAR-T therapy has emerged as a groundbreaking approach, demonstrating unprecedented efficacy in hematological malignancies. CAR-T cells are genetically engineered lymphocytes that express synthetic receptors designed to recognize specific tumor-associated antigens independent of major histocompatibility complex (MHC) presentation [5]. These modified T cells integrate the precision of antibody recognition with the potent cytotoxic capabilities of T lymphocytes, enabling targeted elimination of malignant cells. The remarkable response rates observed in patients with relapsed or refractory B-cell malignancies have led to several FDA-approved CAR-T cell therapies, heralding a new era in cancer immunotherapy [6].

Despite these promising advancements, the translation of CAR-T cell therapy to solid tumors, particularly ovarian cancer, has encountered substantial obstacles. A primary impediment to effective CAR-T cell therapy in ovarian cancer is the complex and immunosuppressive tumor microenvironment (TME) [7]. The ovarian cancer TME comprises a heterogeneous assembly of cellular and non-cellular components, including neoplastic cells, immune cells (such as tumor-associated macrophages, regulatory T cells, and myeloid-derived suppressor cells), stromal cells (including cancer-associated fibroblasts), vascular elements, extracellular matrix, and various soluble factors (cytokines, chemokines, and growth factors) [8, 9]. This intricate milieu exerts profound effects on CAR-T cell trafficking, persistence, and effector functions, ultimately compromising therapeutic efficacy.

**Fig. 1:**
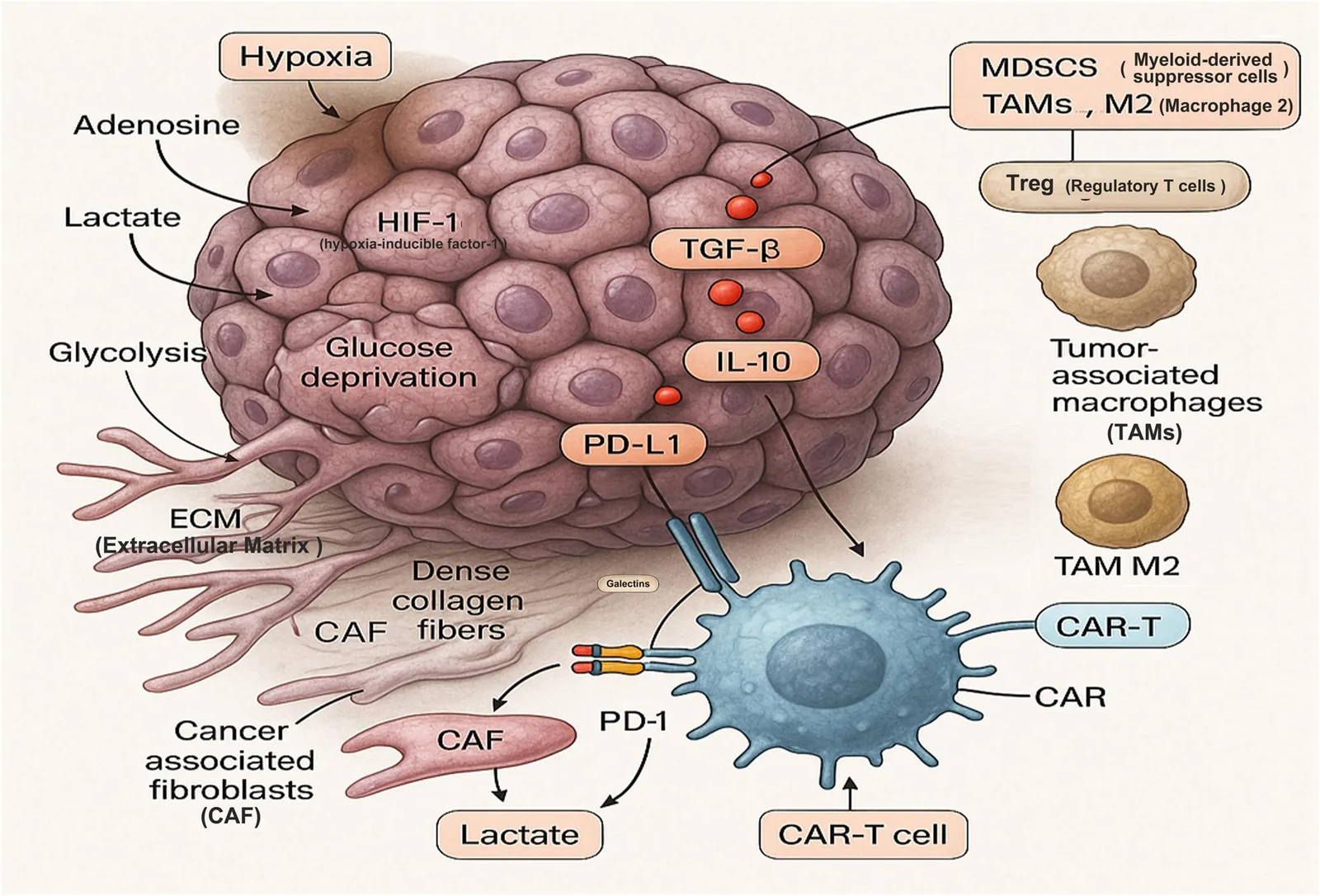
Immunosuppressive tumor microenvironment in ovarian cancer limiting CAR-T cell function. The ovarian tumor microenvironment comprises stromal barriers, immunosuppressive cells including regulatory T cells (Tregs), M2 macrophages, and myeloid-derived suppressor cells (MDSCs), together with inhibitory ligands (e.g., PD-L1, galectins) and cytokines such as transforming growth factor-beta (TGF-β), collectively restricting CAR-T cell infiltration, persistence, and cytotoxic activity

The immunosuppressive mechanisms within the ovarian cancer TME are multifaceted and involve complex cellular and molecular interactions. Central to these mechanisms are immunosuppressive cytokines such as transforming growth factor-beta (TGF-β) and interleukin-10 (IL-10), which directly inhibit T cell proliferation, cytokine production, and cytotoxic activity [10, 11]. Additionally, immunoregulatory cells, including regulatory T cells (Tregs) and myeloid-derived suppressor cells (MDSCs), actively suppress anti-tumor immune responses through various mechanisms such as the production of inhibitory cytokines, consumption of essential metabolites, and direct cell-cell interactions [12]. Understanding the extent to which these cellular and molecular components of the TME can suppress the cytotoxic function of CAR-T cells represents a critical research question that needs comprehensive examination. The complex immunosuppressive landscape of the ovarian cancer TME presents formidable challenges to effective CAR-T cell therapy implementation (Fig. 1). This sophisticated microenvironment encompasses multiple cellular and molecular components that collaboratively suppress anti-tumor immunity while promoting cancer progression. As illustrated in Fig. 1, the spatial organization of immunosuppressive elements creates distinct zones that restrict CAR-T cell infiltration, compromise effector function, and limit therapeutic efficacy, necessitating innovative approaches to overcome these barriers.

Furthermore, the ovarian cancer TME harbors multiple signaling pathways that impede CAR-T cell infiltration and persistence within the tumor mass. Immune checkpoint pathways, particularly the programmed death-1 (PD-1)/programmed death-ligand 1 (PD-L1) and cytotoxic T-lymphocyte-associated protein 4 (*CTLA-4*) axes, induce T cell exhaustion and apoptosis through distinct molecular mechanisms: PD-1 engagement inhibits proximal TCR signaling via SHP-2 phosphatase recruitment, while *CTLA-4* competitively binds CD80/CD86, preventing costimulatory CD28 signaling necessary for T cell activation [13, 14]. Additionally, aberrant vascular architecture and extracellular matrix components present physical barriers to T cell infiltration. Elucidating the signaling patterns in the TME associated with reduced CAR-T cell penetration or persistence constitutes another fundamental research question that warrants detailed investigation [15].

This review addresses critical research questions by examining how the TME regulates CAR-T cell function in ovarian cancer. We discuss the cellular and molecular components of the ovarian cancer TME that inhibit CAR-T cell cytotoxicity, emphasizing immunosuppressive mechanisms mediated by TGF-β, IL-10, Tregs, and MDSCs. Our examination evaluates signaling patterns associated with reduced CAR-T cell infiltration or persistence in tumor masses, focusing on pathways including PD-1/PD-L1, *CTLA-4*, and metabolic pathways such as IDO and hypoxia-induced signaling. Moreover, we assess emerging strategies to overcome these TME-mediated barriers, including advanced CAR-T cell engineering approaches, combinatorial therapeutic strategies, and novel TME-modulating agents. By synthesizing current understanding with innovative approaches, we aim to inform the development of more effective CAR-T cell-based immunotherapies, potentially transforming the therapeutic landscape for this challenging malignancy.

## Tumor microenvironment (TME) in ovarian cancer

The tumor microenvironment in ovarian cancer constitutes a sophisticated biological ecosystem characterized by intricate interactions between malignant cells and diverse stromal components that collectively orchestrate tumor progression, therapeutic resistance, and metastatic dissemination [16, 17]. Rather than functioning as a passive scaffold, the TME actively participates in reciprocal molecular dialogues with neoplastic cells, fostering conditions that promote malignant growth while simultaneously suppressing anti-tumor immunity [18].

### Cellular components of the ovarian cancer TME

#### Cancer-Associated Fibroblasts (CAFs)

Cancer-associated fibroblasts represent a predominant cellular constituent within the ovarian cancer TME [19], secreting a repertoire of growth factors (EGF, FGF, HGF), extracellular matrix proteins, and chemokines (notably CXCL12) that synergistically enhance tumor proliferation, invasion, and metastatic potential while simultaneously conferring resistance to conventional therapies through paracrine signaling and physical barrier formation [20–22].

#### Immunosuppressive immune cells

The immunological milieu of ovarian cancer is characterized by profound immunosuppression mediated through multiple cellular and molecular mechanisms.

*Regulatory T cells (Tregs)*: Regulatory T cells infiltrate ovarian tumors in substantial numbers [23], executing immunoinhibitory functions via secretion of TGF-β and IL-10, competitive consumption of IL-2, and expression of checkpoint molecules including *CTLA-4* [24]. Notably, these immunosuppressive populations are often enriched in ovarian cancer tissues and ascites, where their relative abundance correlates with elevated levels of IL-10, TGF-β, and other inhibitory cytokines, contributing to disease progression and poor clinical outcomes [25].

*Myeloid-Derived Suppressor Cells (MDSCs)*:oncurrently, myeloid-derived suppressor cells compromise T cell functionality through elaboration of reactive oxygen species, nitric oxide, depletion of critical amino acids, and immunoregulatory cytokines [26]. Importantly, MDSCs exhibit functional heterogeneity across monocytic and granulocytic subsets, contributing to distinct immunosuppressive mechanisms within the tumor microenvironment.

*Intratumoral neutrophils*: In addition, intratumoral neutrophils can promote immunosuppression through the release of neutrophil extracellular traps (NETs), which impair T cell function and facilitate tumor progression [27].

*Tumor-Associated Macrophages (TAMs)*: Tumor-associated macrophages exhibiting a predominantly M2-polarized phenotype [28] contribute significantly to the immunosuppressive landscape through production of angiogenic factors (VEGF), matrix-remodeling enzymes (MMPs), and immunoregulatory mediators (IL-10, TGF-β, PGE2), with their abundance correlating with advanced disease stage and inferior survival outcomes [29, 30].

*Tertiary lymphoid structures (TLS)*: In contrast to immunosuppressive populations, B cells organized within tertiary lymphoid structures are associated with favorable prognostic outcomes in ovarian cancer. These structures support local immune activation and are increasingly recognized as functional niches that enhance antitumor immunity rather than suppress it [27].

### Soluble factors and cytokine milieu

#### Immunosuppressive cytokines

The soluble component of the ovarian cancer TME encompasses a complex array of cytokines, chemokines, growth factors, and metabolites that collectively establish a microenvironment conducive to tumor progression while antagonizing anti-tumor immunity [31]. TGF-β and IL-10, present at elevated concentrations in ovarian cancer ascites and tumor tissue, exert pleiotropic effects across multiple cellular compartments within the TME [32].

#### Chemokines and cellular crosstalk

**Fig. 2:**
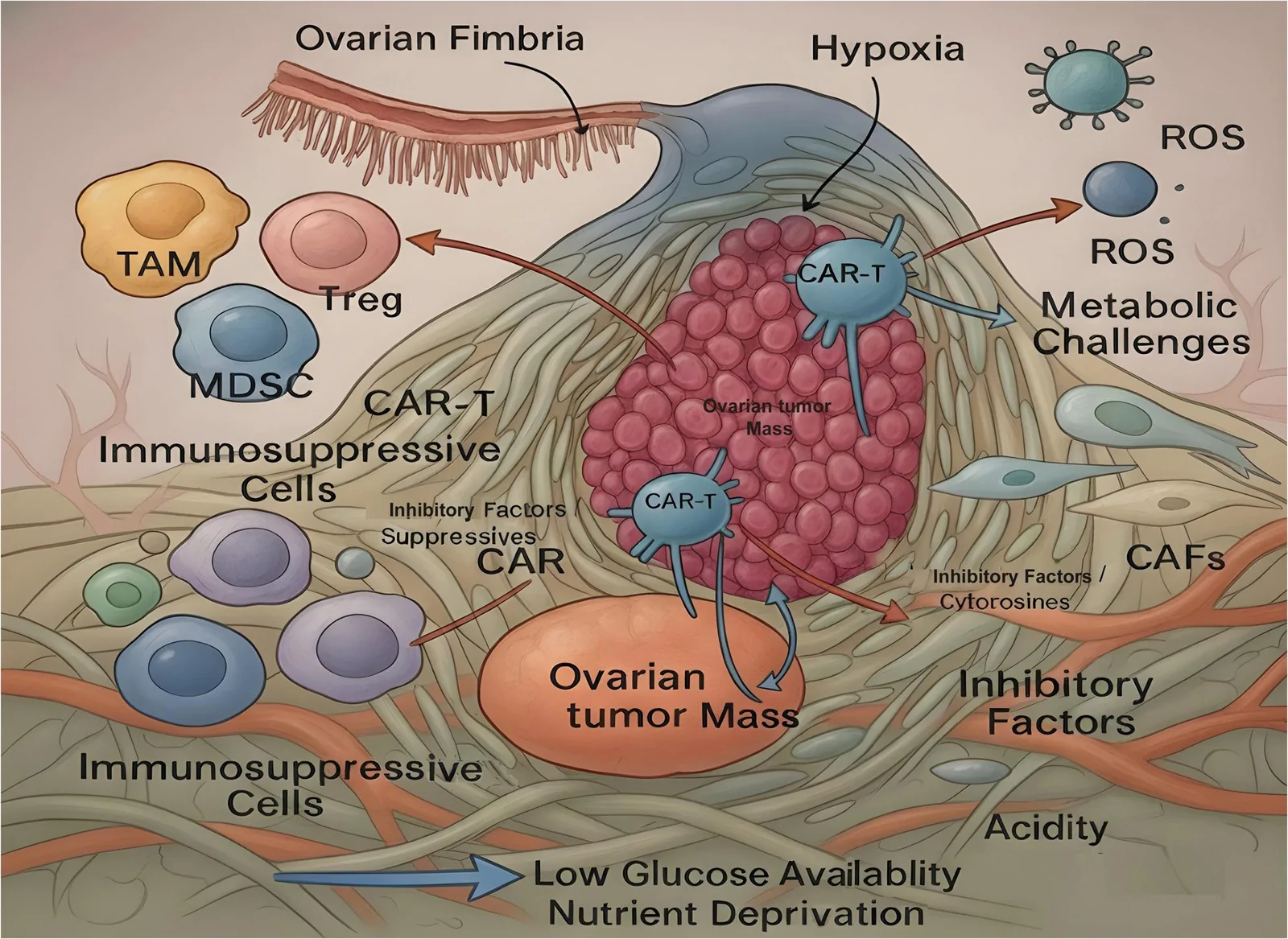
Bidirectional crosstalk between CAR-T cells and immunosuppressive populations in the TME in ovarian cancer. Dynamic signaling interactions between CAR-T cells and tumor-resident immune and stromal populations, including regulatory T cells (Tregs), tumor-associated macrophages (TAMs), and cancer-associated fibroblasts (CAFs), establish feedback networks that reinforce immunosuppression and compromise therapeutic activity

The interactions between tumor-infiltrating immune populations and CAR-T cells significantly impact therapeutic outcomes in ovarian cancer (Fig. 2). As depicted in Fig. 2, bidirectional signaling networks between engineered T cells and resident immunoregulatory populations, including Tregs, TAMs, and CAFs, establish complex feedback loops that predominantly favor immunosuppression. Understanding these cellular crosstalk mechanisms is essential for developing strategies that can disrupt immunosuppressive networks while enhancing CAR-T cell functionality within the hostile ovarian cancer microenvironment.

### Physical and structural barriers

#### Extracellular matrix (ECM) and desmoplasia

The extracellular matrix composition and architecture within the ovarian cancer TME significantly influence malignant behavior and therapeutic efficacy [33]. Desmoplasia, characterized by excessive deposition and cross-linking of ECM proteins [34], creates formidable physical barriers to immune infiltration while simultaneously providing mechanical and biochemical cues that modulate cancer cell behavior and therapeutic response [35].

#### Abnormal vascular architecture

The vascular network within ovarian tumors exhibits significant structural and functional abnormalities that contribute to disease progression and therapeutic challenges [36].

#### The peritoneal cavity microenvironment

The peritoneal cavity, representing the primary site of ovarian cancer metastasis, constitutes a distinctive microenvironmental niche with unique biological characteristics that influence disease progression and response to therapeutic interventions [37].

### Metabolic and biochemical features

#### Hypoxia and HIF signaling

Hypoxia emerges as a fundamental characteristic of the ovarian cancer TME, triggering adaptive responses predominantly mediated by hypoxia-inducible factors (HIFs) [38]. In malignant cells, hypoxia promotes metabolic reprogramming toward glycolysis, enhances invasive capacity, and confers resistance to apoptosis and therapeutic interventions [39]. In infiltrating lymphocytes, oxygen deprivation compromises proliferative potential, cytokine elaboration, and cytolytic function [40].

#### Metabolic competition and nutrient depletion

The metabolic landscape of the ovarian cancer TME is characterized by significant alterations that pose substantial challenges for infiltrating immune cells. Cancer cells exhibit enhanced glycolysis even in oxygen-replete conditions (Warburg effect) [41], creating a glucose-depleted, lactate-enriched environment that profoundly impairs lymphocyte functionality and contributes to immune evasion [42].

### TME-mediated metastasis and therapeutic resistance

The TME plays a pivotal role in facilitating ovarian cancer metastasis, which occurs predominantly through transcoelomic dissemination within the peritoneal cavity [43]. Malignant cells detach from primary tumors, frequently as multicellular spheroids exhibiting enhanced anoikis resistance, and disseminate throughout the peritoneal cavity via ascitic fluid [44].

Furthermore, the TME significantly contributes to therapeutic resistance in ovarian cancer—a formidable clinical challenge underpinning the high recurrence rates and poor survival outcomes characteristic of this malignancy [45]. The dynamic and reciprocal interactions between malignant cells and stromal components establish self-reinforcing feedback loops that perpetuate and amplify the malignant phenotype [46].

Contemporary therapeutic paradigms increasingly recognize the critical importance of targeting not only neoplastic cells but also the supportive microenvironment, disrupting the symbiotic relationships that drive disease progression. This integrative approach holds substantial promise for improving clinical outcomes in ovarian cancer, a malignancy characterized by therapeutic recalcitrance and poor long-term survival.

## CAR-T cells: mechanism, design, and current status

### Historical development and conceptual foundation

Chimeric Antigen Receptor T (CAR-T) cell therapy represents a revolutionary approach in cancer immunotherapy that harnesses the specificity of antibody recognition combined with T lymphocyte cytotoxicity [47]. Since its conceptual foundation established by Eshhar and colleagues in the late 1980s, this therapeutic modality has evolved from demonstrating that T cells could be engineered to recognize antigens independent of MHC restriction to achieving breakthrough clinical successes in hematological malignancies [48]. CAR-T cell therapy represents a paradigm shift in cancer treatment by combining the specificity of antibody recognition with the cytotoxic potential of T lymphocytes, enabling targeted elimination of malignant cells while potentially overcoming limitations of conventional therapies [49]. The evolution of this technology from conceptual framework to clinical application highlights both its revolutionary potential and the ongoing challenges in adapting this approach to solid tumors, particularly ovarian cancer [50].

### Structural architecture of CAR molecules

#### Evolution from first to fifth generation CARs

The structural architecture of CAR molecules has undergone significant refinement through multiple generations [51]. First-generation CARs incorporated only the CD3ζ signaling domain, resulting in limited clinical efficacy due to insufficient costimulation [52]. Second-generation CARs introduced additional costimulatory domains (CD28 or 4-1BB), significantly enhancing T cell proliferation, cytokine production, and persistence [53]. Third-generation designs incorporate multiple costimulatory domains to provide complementary activation signals, though clinical advantages over second-generation constructs remain under investigation [54]. Fourth-generation “armored” CARs feature additional genetic modifications enabling expression of immunomodulatory cytokines or functional proteins designed to reshape the tumor microenvironment [55]. The emerging fifth-generation CARs incorporate STAT3/5 signaling domains derived from cytokine receptors, potentially enhancing proliferation and persistence in immunosuppressive environments [56].

#### Signaling domains and costimulatory molecules

CD28-based constructs typically yield rapid expansion with shorter persistence, while 4-1BB-containing CARs demonstrate sustained persistence with more measured activation kinetics [53].

### Mechanism of action and tumor cell elimination

The mechanism of action begins with CAR recognition of target antigens expressed on malignant cells, a process that occurs independent of antigen processing or MHC presentation [57]. Antigen engagement initiates a signaling cascade involving immunoreceptor tyrosine-based activation motifs within the CD3ζ domain, recruitment and activation of ZAP70 kinase, and subsequent activation of multiple downstream pathways including MAPK, PI3K/AKT, and NF-κB [58]. Activated CAR-T cells eliminate tumor targets through perforin and granzyme exocytosis, death receptor engagement, inflammatory cytokine production, and induction of secondary immune responses [59].

### Manufacturing process and quality control

Manufacturing clinical-grade CAR-T cells involves leukapheresis, T cell isolation and activation, genetic modification using viral vectors or non-viral approaches, ex vivo expansion, quality assessment, cryopreservation, and infusion following lymphodepleting chemotherapy [60]. Recent innovations focus on optimizing T cell subsets for modification, developing closed-system automated platforms, implementing novel culture conditions, and exploring allogeneic approaches using gene-editing technologies.

### CAR-T Therapy in hematological malignancies

Clinical implementation of CAR-T cell therapy has achieved transformative outcomes in patients with B-cell lymphomas and acute lymphoblastic leukemia, with multiple constructs receiving regulatory approval from the FDA and EMA. These therapeutic platforms have demonstrated durable complete remissions in heavily pretreated patients with relapsed or refractory disease, populations historically associated with dismal prognoses and limited treatment options. The therapeutic efficacy in hematological malignancies stems from favorable biological characteristics including homogeneous antigen expression across malignant cell populations, unrestricted access of engineered T cells to circulating tumor cells, and relatively permissive immune environments that facilitate robust CAR-T cell expansion and sustained persistence [6].

### Translation to solid tumors

While achievements in hematological contexts validate the CAR-T principle, extension to solid malignancies confronts distinct biological impediments. Ovarian cancer exemplifies these challenges through its characteristically hostile microenvironment that actively suppresses therapeutic T cell function [61]. The complex interplay between physical barriers, metabolic constraints, and immunoregulatory networks within ovarian tumors creates a multidimensional obstacle to CAR-T efficacy. Unlike circulating malignancies, solid tumor architecture imposes spatial restrictions on T cell trafficking while establishing biochemical gradients of immunosuppressive factors that progressively attenuate CAR-T cell activation and cytotoxic capacity [7].

### Target antigens in ovarian cancer

Several target antigens have been investigated specifically for ovarian cancer, including mesothelin (overexpressed in approximately 70% of cases), MUC16 (CA-125), folate receptor-α, HER2, and B7-H3. Importantly, the heterogeneity of antigen expression and the risk of on-target off-tumor toxicity remain key challenges in selecting optimal CAR-T targets in ovarian cancer [62–64].

#### Mesothelin

Mesothelin-directed CAR-T cells have demonstrated modest anti-tumor activity with manageable toxicity profiles, though durable responses remain elusive [65].

#### MUC16 (CA-125)

Innovative approaches targeting MUC16 incorporate IL-12 secretion capabilities to enhance activity within the immunosuppressive tumor microenvironment [66].

#### Folate receptor-α and other potential targets

Additional targets including folate receptor-α, HER2, and B7-H3 have shown promise in preclinical investigations [67].

### Clinical trials in ovarian cancer: current status

Clinical studies have revealed CAR-T cell trafficking to tumor sites (though often with limited infiltration), transient anti-tumor activity without complete responses, generally manageable safety profiles, and rapid CAR-T cell functional impairment in the tumor microenvironment [68].

These challenges have prompted development of next-generation approaches specifically designed to address solid tumor barriers, including dual or tandem CARs targeting multiple antigens, armored CAR-T cells engineered to express immunomodulatory molecules, combinatorial approaches with checkpoint inhibitors, localized administration strategies, and logic-gated CAR systems requiring multiple activation signals [69].

### Toxicity profiles and management

#### Cytokine Release Syndrome (CRS)

CAR-T cellular immunotherapy exhibits unique toxicity signatures demanding specialized clinical oversight. Cytokine release syndrome manifests following robust T-cell activation and subsequent inflammatory mediator cascade. Management strategies commonly include tocilizumab (IL-6 receptor blockade) and corticosteroids, depending on severity and clinical presentation [70].

#### Immune Effector Cell-Associated Neurotoxicity (ICANS)

Immune effector cell-associated neurotoxicity syndrome (ICANS) presents as encephalopathy with variable neurological manifestations. Corticosteroids remain the primary management strategy, while tocilizumab may be used in cases with concurrent CRS [71].

#### On-target, off-tumor effects

On-target, off-tumor effects may occur when target antigens are expressed on non-malignant tissues [72]. Standardized grading systems, early intervention strategies, and effective management approaches—including tocilizumab for CRS and corticosteroids for ICANS—have significantly improved safety profiles [73].

The evolving landscape of CAR-T cell therapy for ovarian cancer encompasses several promising research trajectories: integration of advanced synthetic biology approaches, exploitation of gene-editing technologies, development of allogeneic platforms, identification of novel target antigens, engineering strategies specifically addressing the unique challenges of the ovarian cancer microenvironment, and refinement of patient selection strategies [51, 74].

**Fig. 3:**
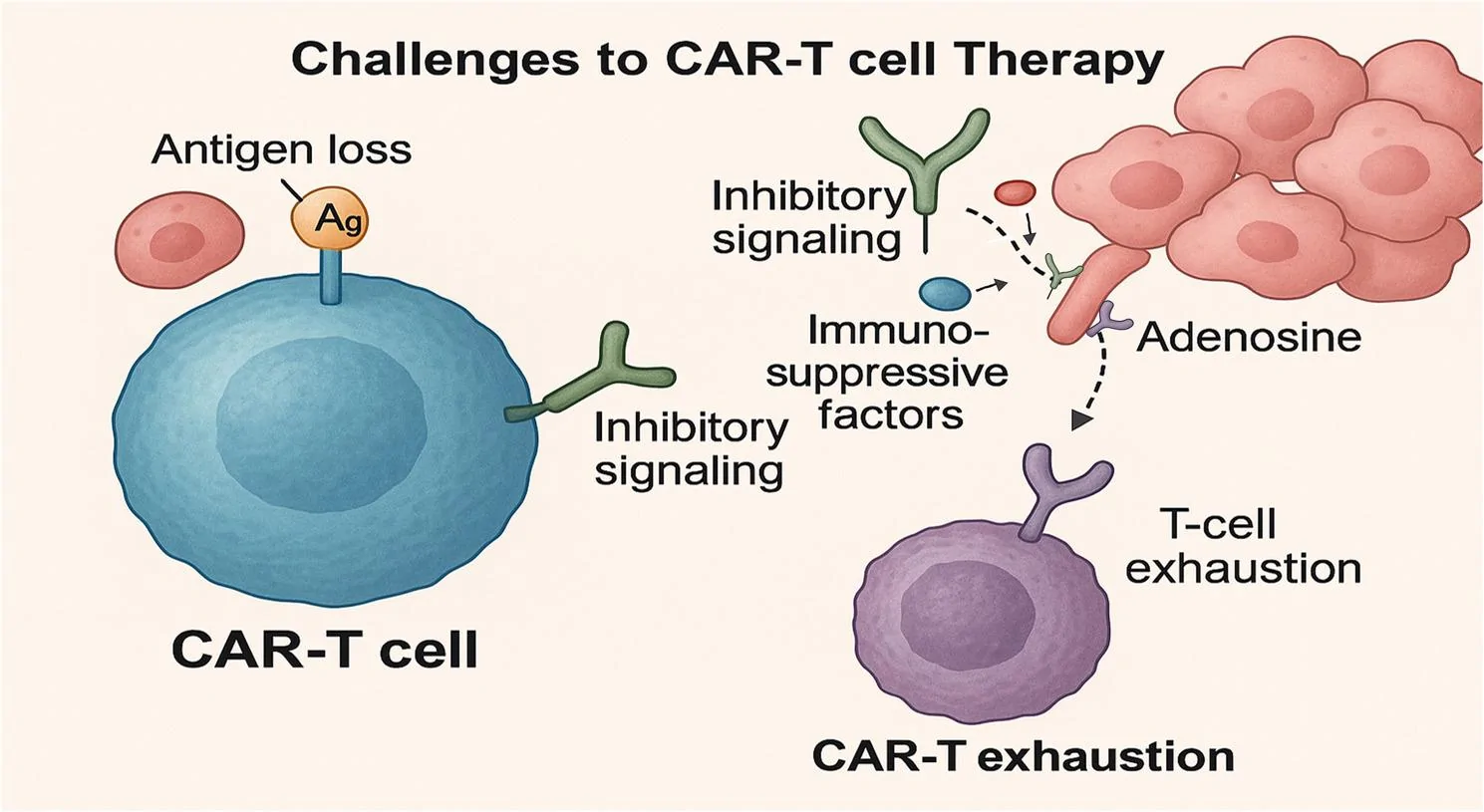
Multidimensional resistance mechanisms limiting CAR-T cell efficacy in ovarian cancer. Therapeutic resistance arises from coordinated processes including antigen escape, upregulation of inhibitory checkpoint pathways, metabolic dysregulation, and physical exclusion by stromal barriers, which collectively impair CAR-T cell persistence and antitumor function

Despite significant obstacles, ongoing technological innovations and deeper understanding of tumor-immune interactions continue to expand the therapeutic frontier of CAR-T cell therapy for ovarian cancer [75]. The continued evolution of CAR design, manufacturing processes, and combinatorial strategies offers promising avenues to address the unique challenges imposed by the ovarian tumor microenvironment, potentially transforming treatment paradigms for this challenging gynecological malignancy [76]. Despite the revolutionary potential of CAR-T cell therapy, multiple resistance mechanisms limit its efficacy in solid tumors, particularly ovarian cancer (Fig. 3). As summarized in Fig. 3, these barriers encompass diverse biological processes including antigen escape, immune checkpoint upregulation, metabolic dysregulation, and exclusion via physical barriers. These resistance pathways operate simultaneously to create a multidimensional challenge that necessitates comprehensive approaches addressing multiple aspects of the tumor-immune interface to achieve durable therapeutic responses. In parallel, CAR-engineered natural killer T (CAR-NKT) cells have emerged as a complementary strategy with distinct immunological properties. These cells can recognize lipid antigens presented by CD1d and exert both innate and adaptive immune functions. Notably, CAR-NKT cells have demonstrated the capacity to modulate the immunosuppressive tumor microenvironment by reducing tumor-associated macrophages and myeloid-derived suppressor cells, thereby enhancing antitumor responses in solid tumors, including ovarian cancer [77].

## Challenges in TME for CAR-T therapy in ovarian cancer

TME in ovarian cancer presents formidable barriers to effective CAR-T cell therapy implementation. This complex immunosuppressive network significantly impairs CAR-T cell function, persistence, and tumor infiltration capabilities through multiple interconnected mechanisms.

### Immune evasion mechanisms in ovarian cancer TME

Ovarian cancer employs sophisticated immune evasion strategies that fundamentally challenge CAR-T efficacy. PD-L1 expression on both malignant cells and stromal components engages with PD-1 receptors on infiltrating CAR-T cells, suppressing PI3K/Akt pathway activation and diminishing cytotoxic potential [78]. Comprehensive transcriptomic analyses have revealed upregulation of multiple checkpoint receptors beyond PD-1, including *CTLA-4*, LAG-3, TIM-3, and TIGIT, creating a multi-layered immunosuppressive landscape with expression patterns that intensify with disease progression and contribute to treatment resistance [79].

The ovarian cancer TME harbors diverse immunosuppressive cell populations. Regulatory T cells (Tregs) secrete IL-10, IL-35, and TGF-β, creating a hostile environment for CAR-T activity [79]. Single-cell RNA sequencing demonstrates that ovarian cancer Tregs exhibit enhanced suppressive capacity compared to peripheral Tregs [80]. This increased suppressive function is associated with elevated expression of immunosuppressive genes such as *FOXP3*, *CTLA4*, *IL2RA* (*CD25*), and *TGF-β1*, as revealed by the transcriptional profiling [81]. In addition, tumor-infiltrating Tregs show upregulation of exhaustion-related markers and metabolic genes that support their stability and function within the hostile tumor microenvironment. These features suggest a transcriptional reprogramming of Tregs in the tumor milieu that reinforces their immunosuppressive phenotype [82]. Additionally, myeloid-derived suppressor cells (MDSCs) exert multifaceted suppressive effects through amino acid depletion, reactive oxygen species production, and immunomodulatory factor secretion [83]. Proteomic analyses have identified unique MDSC secretomes enriched in S100A8/A9 and prostaglandin E2 that directly impair T cell receptor signaling and metabolic fitness [84].

### Barriers to CAR-T Cell trafficking and infiltration

Physical and biochemical barriers within ovarian cancer TME significantly restrict CAR-T cell trafficking and penetration. Dysregulated vascular architecture within neoplastic tissues impedes CAR-T migration from circulation, as intravital microscopic analyses validate that vascular competence in tumors fundamentally influences therapeutic efficacy [85]. The dense extracellular matrix (ECM), particularly enriched in collagen, fibronectin, and hyaluronic acid, forms a physical barrier limiting CAR-T cell mobility. Proteomic analysis reveals a unique ECM composition with high concentrations of versican and decorin that specifically impede T cell motility [86].

Chemokine-receptor mismatches further compromise CAR-T cell trafficking. While activated T cells typically express CXCR3, CCR5, and CCR2, ovarian cancer tissue often exhibits reduced expression of their corresponding ligands, resulting in inadequate chemotactic gradients [87]. Transcriptomic analyses have shown that ovarian cancers uniquely downregulate CXCL9 and CXCL10 through epigenetic silencing. Simultaneously, ovarian cancer cells secrete alternative chemokines like CXCL12 that preferentially attract immunosuppressive cell populations [88].

### Biochemical and metabolic constraints

The metabolic landscape within the ovarian cancer TME profoundly affects CAR-T functionality through multiple mechanisms. Hypoxia, a prominent feature of solid tumors, induces numerous immunosuppressive pathways through HIF-1α stabilization [89]. Metabolomic profiling has revealed extreme oxygen tension gradients that correlate with distinct zones of T cell dysfunction within tumor masses. Under these hypoxic conditions, CAR-T cells demonstrate significantly impaired cytotoxicity and proliferative capacity due to metabolic reprogramming toward less efficient glycolytic pathways [90].

Competition for essential nutrients, particularly glucose, further compromises CAR-T cell energetics. Ovarian cancer cells characteristically display upregulated glucose transporters and enhanced glycolytic capacity, creating a competitive advantage over therapeutic T cells [91]. Metabolic tracing studies using isotope-labeled glucose have demonstrated preferential uptake by tumor cells compared to infiltrating lymphocytes, effectively creating ‘metabolic starvation’ conditions for therapeutic CAR-T cells in the tumor microenvironment [92].

The glycolytic shift characteristic of ovarian cancer cells generates excessive lactate accumulation that establishes a profoundly immunosuppressive microenvironment. Tumor-derived lactate, exported through monocarboxylate transporters (MCT1/MCT4), acidifies the extracellular milieu while simultaneously serving as a signaling molecule that impairs CAR-T cell function through multiple mechanisms. Elevated lactate concentrations inhibit T cell glycolytic capacity by competing for MCT1-mediated pyruvate export, disrupt intracellular pH homeostasis, suppress nuclear factor of activated T cells (NFAT) signaling, and promote expression of immunosuppressive genes through lactylation of histone proteins. Furthermore, lactate-rich conditions preferentially support Treg proliferation and M2 macrophage polarization, creating a metabolically hostile niche that simultaneously starves effector CAR-T cells while enhancing immunoregulatory cell populations [93].

Additional metabolic abnormalities include increased indoleamine 2,3-dioxygenase (IDO) activity, which depletes tryptophan while generating immunosuppressive kynurenine metabolites [94]. Comprehensive metabolomic analyses have identified elevated kynurenine/tryptophan ratios that inversely correlate with tumor-infiltrating lymphocyte functionality [95]. Cancer-associated fibroblasts demonstrate upregulated expression of arginase and inducible nitric oxide synthase (iNOS), depleting arginine and generating nitric oxide that further suppresses T cell signaling [96].

Adenosine accumulation within the ovarian cancer TME represents an additional immunometabolic checkpoint that profoundly suppresses CAR-T cell function. Hypoxia and metabolic stress induce extracellular ATP release from dying tumor cells, which is rapidly converted to adenosine by ectonucleotidases CD39 and CD73 highly expressed on tumor cells, stromal fibroblasts, and immunosuppressive myeloid populations. Elevated adenosine levels engage A2A and A2B receptors on infiltrating CAR-T cells, triggering cAMP-dependent signaling cascades that inhibit T cell receptor activation, suppress cytokine production, and promote expression of exhaustion markers. This purinergic signaling axis creates a feed-forward immunosuppressive loop, as adenosine simultaneously enhances Treg suppressive capacity while impairing effector T cell cytotoxicity, representing a critical barrier to sustained CAR-T therapeutic efficacy [97].

### Genetic and epigenetic mechanisms

Genetic adaptations in ovarian cancer cells directly counter T cell-mediated immunity. Loss of antigen presentation machinery has been documented in 30–40% of high-grade serous ovarian carcinomas. While CAR-T cells function independently of MHC presentation, heterogeneous expression of target antigens presents another challenge [98]. Multi-region sequencing has revealed substantial intratumoral heterogeneity in common CAR-T targets like MUC16, mesothelin, and folate receptor alpha, allowing for immune escape through antigen-negative variants [62].

### Clinical and experimental evidence of TME-mediated CAR-T resistance

Clinical data from early-phase trials provide crucial insights into resistance mechanisms. In a phase I trial evaluating mesothelin-targeted CAR-T cells, post-treatment biopsies revealed limited infiltration despite peripheral persistence, with prominent PD-1 expression on infiltrating CAR-T cells. Single-cell analyses demonstrated spatial correlation between high TGF-β expression areas and zones lacking T cell infiltration [65].

Patient-derived xenograft models treated with MUC16-targeted CAR-T cells showed initial tumor recognition but rapid CAR-T dysfunction characterized by progressive loss of cytokine production and cytotoxic capacity. Transcriptomic profiling revealed a distinct exhaustion signature with upregulation of inhibitory receptors and metabolic stress response genes [99].

Three-dimensional in vitro models have demonstrated how spatial organization influences CAR-T function, revealing efficient cancer cell elimination at spheroid peripheries but failure to penetrate deeply. Time-lapse imaging with multiplexed cytokine analysis has documented direct inhibitory cell-to-cell contacts and localized gradients of inhibitory mediators [100].

Comparative analyses between responders and non-responders have identified specific TME signatures associated with treatment failure. Mass cytometry analysis of resistant murine ovarian tumors revealed enrichment of polymorphonuclear MDSCs expressing high levels of arginase and iNOS [101]. Spatial transcriptomics of human samples has identified distinct immunosuppressive niches characterized by colocalization of cancer-associated fibroblasts secreting CXCL12 alongside CXCR4-expressing Tregs [102].

**Fig. 4:**
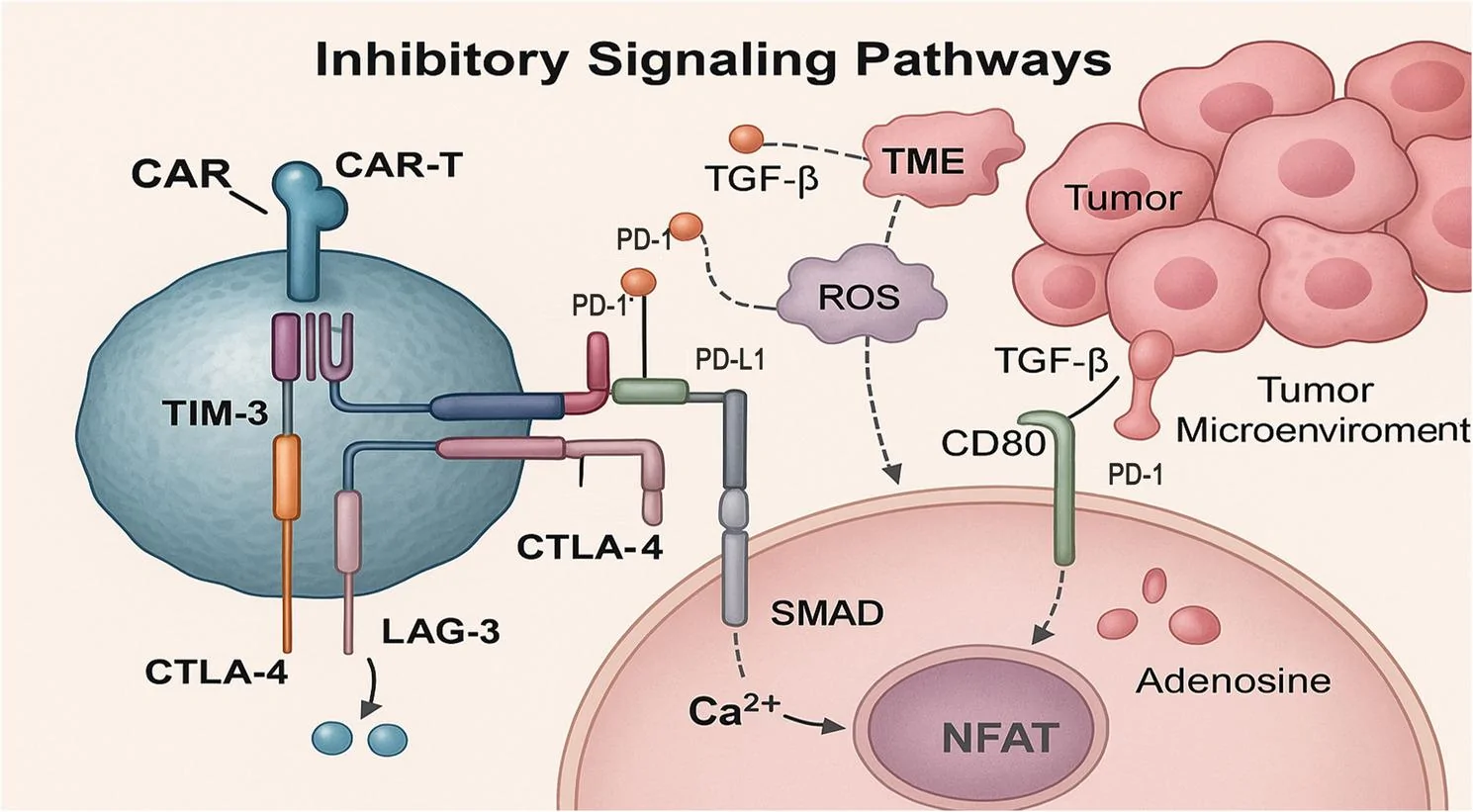
Inhibitory signaling pathways within the tumor microenvironment impairing CAR-T cell function. The schematic depicts key inhibitory signaling axes within the tumor microenvironment (TME), including PD-1/PD-L1 interactions, TGF-β signaling, adenosine-mediated suppression, and immune checkpoints such as LAG-3 (Lymphocyte activation gene 3) and CTLA-4(Cytotoxic T-lymphocyte associated protein 4). These pathways converge through intracellular mediators, including Ca²⁺ flux, ROS (Reactive oxygen species), TIM-3 (T-cell immunoglobulin mucin-3), SMAD signaling, and NFAT (nuclear factor of activated T cells) dysregulation, ultimately attenuating CAR-T cell activation, signaling capacity, and effector function

The multifaceted nature of TME-mediated immunosuppression to CAR-T therapy in ovarian cancer necessitates comprehensive strategies addressing multiple aspects of the immunosuppressive microenvironment simultaneously. Despite promising advances, current CAR-T strategies remain limited by tumor heterogeneity, antigen escape, and insufficient persistence, highlighting the need for more refined and context-specific therapeutic approaches. Integration of advanced imaging with molecular profiling continues to reveal increasingly detailed mechanisms of CAR-T failure, informing the development of next-generation therapeutic approaches designed to overcome these specific barriers [103]. Innovative CAR-T cell engineering strategies have emerged to specifically address the challenges presented by the ovarian cancer microenvironment (Fig. 4). As illustrated in Fig. 4, these approaches include armoring CAR-T cells with immunostimulatory cytokines, integrating switch receptors that convert inhibitory signals into activating ones, enhancing tumor tropism through chemokine receptor engineering, and developing multi-targeted constructs. These sophisticated modifications represent a significant evolution from conventional CAR designs, demonstrating potential to overcome the complex barriers that have historically limited solid tumor applications.

## Novel Strategies to overcome TME-mediated immunosuppression in ovarian cancer CAR-T therapy

Notwithstanding remarkable outcomes in hematologic neoplasms, CAR-T interventions demonstrate restricted effectiveness against ovarian carcinoma, primarily attributable to the immunoinhibitory milieu characterizing its tumor microenvironment. Recent scientific and technological advances have led to the development of innovative strategies designed to enhance CAR-T cell persistence, trafficking, and cytotoxic function within this hostile microenvironment. These approaches target specific aspects of the TME-mediated immunosuppression through genetic engineering, combination therapies, and novel delivery systems that collectively aim to overcome the multifaceted barriers to effective CAR-T cell therapy in solid tumors.

### Genetic engineering of CAR-T Cells for TME resistance

Genetic modification strategies effectively counteract TME-mediated immunosuppression. CRISPR/Cas9-mediated disruption of PD-1 expression in mesothelin-targeting CAR-T cells demonstrates improved anti-tumor activity with prolonged persistence despite exposure to PD-L1-expressing tumor cells and immunosuppressive cytokines [104, 105].

Engineering CAR-T cells to express dominant-negative TGF-β receptors confers resistance to TGF-β-mediated inhibition, maintaining proliferative capacity and cytotoxic function. These modified cells sustain expression of activation markers and produce higher levels of pro-inflammatory cytokines in TGF-β-rich environments characteristic of ovarian tumors [106].

“Armored” CAR-T cells constitutively expressing stimulatory cytokines (IL-12, IL-15, or IL-18) exhibit enhanced TME-resistance by creating favorable immune microenvironments while simultaneously recruiting endogenous immune cells. In ovarian cancer xenograft models, IL-12-secreting CAR-T cells demonstrate superior tumor infiltration and persistence, resulting in improved tumor control and survival outcomes [55, 107].

### Combination therapies with immune checkpoint inhibitors

The synergistic potential of combining CAR-T therapy with immune checkpoint inhibitors represents a promising strategy. Clinical trials evaluating mesothelin-targeted CAR-T cells with pembrolizumab (anti-PD-1) demonstrate encouraging preliminary results, with improved CAR-T cell expansion and persistence in recurrent ovarian cancer patients [108].

Preclinical studies exploring CAR-T cells with dual checkpoint inhibition (targeting both PD-1 and *CTLA-4*) show enhanced anti-tumor activity compared to either CAR-T cells alone or with single checkpoint inhibition. Dual blockade creates a more permissive TME by simultaneously mitigating multiple immunosuppressive mechanisms [109].

Recent investigations focus on novel checkpoint inhibitors targeting TME-specific pathways (LAG-3, TIM-3, and TIGIT) particularly relevant in ovarian cancer. Preliminary data suggest these alternative checkpoint inhibitions may complement PD-1/PD-L1 blockade, providing more comprehensive reversal of TME-mediated immunosuppression [110]. While combination therapies hold therapeutic promise, their high cost poses a major challenge for clinical adoption. CAR-T cell manufacturing is inherently expensive, and the addition of checkpoint inhibitors further elevates treatment costs. Without predictive biomarkers to identify likely responders, broad implementation may result in limited cost-effectiveness. Economic modeling studies are needed to evaluate long-term outcomes, budget impact, and value-based pricing for such combination regimens.

### Nanoparticle-based delivery systems

Innovative nanoparticle technologies overcome barriers to CAR-T cell delivery and function within ovarian cancer TME. Biodegradable nanoparticles loaded with immunomodulatory agents selectively release cargo within the tumor microenvironment, creating localized immune-supportive niches while minimizing systemic toxicity [111–113].

Liposomal formulations containing PI3K-γ inhibitors effectively reprogram tumor-associated macrophages from immunosuppressive M2 to pro-inflammatory M1 phenotypes by blocking the PI3K-γ-dependent signaling cascade that otherwise promotes immunosuppressive gene expression and cytokine production [114]. When administered with CAR-T therapy, these nanoparticles significantly enhance T cell infiltration and persistence, improving tumor control in preclinical models through complementary immune-activating mechanisms [115].

Polymeric nanoparticles delivering hypoxia-inducible factor inhibitors mitigate hypoxia-induced immunosuppression by normalizing the metabolic landscape within ovarian tumors, creating favorable conditions for CAR-T cell function with improved glucose availability and reduced immunosuppressive metabolites [116].

Advanced nanoparticle platforms enable localized cytokine delivery. Mesoporous silica nanoparticles with sustained IL-2 release enhance CAR-T cell persistence and proliferation without inducing systemic toxicity in murine ovarian cancer models [117]. Despite encouraging preclinical data, the clinical translation of nanoparticle-based systems faces significant barriers. These include regulatory complexities surrounding long-term biocompatibility, immune clearance, and off-target accumulation. Additionally, scalable manufacturing under GMP standards, quality control of particle size and drug loading, and reproducibility across production batches remain unresolved challenges that hinder clinical implementation.

### microbiome engineering approaches

Emerging evidence indicates the gut microbiome significantly influences immune responses in cancer through multiple mechanisms, including modulation of systemic cytokine profiles, regulation of myeloid cell function, and metabolite-mediated epigenetic reprogramming of immune cells. Specific bacterial strains, particularly those producing short-chain fatty acids such as butyrate, enhance memory T cell formation and improve metabolic fitness, potentially influencing CAR-T cell function and persistence beyond the gastrointestinal tract [118].

Microbial-derived metabolites such as butyrate enhance T cell memory formation by promoting histone acetylation and central memory phenotypes. Additionally, modulation of the aryl hydrocarbon receptor (AhR) pathway in myeloid cells boosts dendritic cell activation and antigen presentation, while engineered probiotics may locally suppress immunosuppressive signals via cytokine or nanobody secretion.

Preclinical studies demonstrate that defined bacterial consortia, especially those enriched in Bifidobacterium species, enhance anti-tumor activity by augmenting dendritic cell function and subsequent T cell responses. Combined with CAR-T therapy in ovarian cancer models, these probiotic interventions result in improved tumor control through modulation of systemic inflammatory responses and TME metabolic reprogramming [119].

Engineered probiotics designed to colonize the tumor microenvironment and secrete therapeutic payloads represent an innovative approach to TME modulation. Genetically modified bacteria producing immunostimulatory cytokines or enzymes that degrade immunosuppressive metabolites show preliminary efficacy in preclinical models, as exemplified by Salmonella strains engineered to express and secrete anti-PD-L1 nanobodies [120]. Beyond existing probiotic interventions, future designs may incorporate engineered microbiota programmed to respond to tumor-specific metabolic cues, releasing therapeutic payloads only under hypoxic or immunosuppressive conditions, thus enabling spatially confined immune activation.

### Metabolic reprogramming strategies

The metabolically restrictive ovarian cancer TME impairs CAR-T cell function. Metformin, an AMPK activator and complex I inhibitor, normalizes glucose metabolism within the TME, improving nutrient availability for infiltrating CAR-T cells. Preclinical studies combining metformin with CAR-T therapy show enhanced T cell persistence and improved anti-tumor activity [121].

Engineering CAR-T cells with enhanced metabolic fitness offers another promising approach. CAR-T cells overexpressing carnitine palmitoyltransferase 1a demonstrate improved persistence in nutrient-restricted environments characteristic of ovarian tumors. Similarly, CAR-T cells engineered to express uncoupling protein 2 resist reactive oxygen species-mediated suppression, maintaining functional capacity within the oxidative stress-rich ovarian cancer TME [122].

Targeting tumor-associated fibroblasts, which contribute to immunometabolic dysfunction, shows promise. Strategies employing FAP-targeted CAR-T cells in combination with conventional tumor-targeted CAR-T cells demonstrate synergistic effects, simultaneously targeting tumor cells while remodeling the stromal component to create favorable conditions for immune cell function [123].

### Strategic considerations for translational optimization of CAR-T therapy

Early-phase clinical trials evaluating TME-modulating strategies with CAR-T therapy yield promising results. A phase I trial (NCT03747965) evaluating mesothelin-targeted CAR-T cells demonstrates acceptable safety and preliminary anti-tumor activity in recurrent ovarian cancer [124]. Similarly, a phase I/II study (NCT03030001) investigating NY-ESO-1-targeted T cells with the TGF-β receptor kinase inhibitor galunisertib shows enhanced T cell persistence and encouraging response rates [125, 126].

Despite these promising results, challenges remain in clinical translation. The complex and heterogeneous nature of ovarian cancer TME necessitates personalized approaches, potentially requiring comprehensive profiling of individual patients’ tumor microenvironments. Additionally, potential increased toxicities with combination approaches warrant careful consideration and effective management strategies. Emerging evidence also underscores the importance of combinatorial approaches, such as coupling CAR-T therapy with immune checkpoint blockade or metabolic reprogramming, to overcome TME-mediated immunosuppression mechanisms [127, 128].

Future directions include synthetic biology approaches enabling dynamic sensing and response to TME conditions. CAR-T cells with synthetic gene circuits capable of detecting specific TME signals represent a promising frontier in adaptive immunotherapy. Future platforms may integrate biosensing elements enabling CAR-T cells to dynamically modulate their effector functions in real-time, adapting to fluctuating TME conditions such as pH, ROS, or cytokine gradients. Integration of artificial intelligence and machine learning algorithms for predicting optimal combinations of TME-modulating strategies based on patient-specific data may facilitate more effective personalized treatment approaches [129, 130]. Achieving optimal therapeutic outcomes in ovarian cancer requires multifaceted approaches that simultaneously address various aspects of tumor-immune interactions. These strategies encompass targeting multiple tumor antigens to mitigate escape mechanisms, neutralizing inhibitory checkpoint pathways, engineering constitutive cytokine production to sustain activation in hostile environments, and optimizing CAR architecture to enhance signaling capacity and persistence. The integration of these complementary approaches offers unprecedented potential to transform CAR-T therapy from a promising concept to clinical reality for patients with recalcitrant ovarian malignancies. Recent advances highlight the role of artificial intelligence in optimizing CAR design, identifying novel targets, and enabling precise TME profiling for improved patient stratification, including patient-specific antigen selection and adaptive CAR design strategies, and therapeutic outcomes [131].

## Future perspectives and clinical translation

The progressive refinement of CAR-T strategies targeting ovarian neoplasia constitutes an emerging domain within immunoncology, harboring substantial capacity to revolutionize therapeutic frameworks. Despite encouraging advances, substantial challenges remain in translating preclinical success to clinical efficacy. Recent studies have further highlighted innovative strategies to enhance CAR-T cell efficacy in ovarian cancer, including the integration of multi-targeted CAR constructs and modulation of the tumor microenvironment to improve persistence and infiltration [132].

### Emerging trends in solid tumor CAR-T development

Next-generation CAR-T therapies address unique challenges presented by solid tumors. Multi-targeted CAR-T cells recognizing multiple tumor-associated antigens simultaneously mitigate antigen escape mechanisms in heterogeneous ovarian tumors. Dual or tandem CAR constructs targeting combinations such as mesothelin/MUC16 or HER2/folate receptor alpha demonstrate enhanced tumor recognition and reduced escape variant emergence in preclinical models [62, 133]. An emerging concept involves integration of tumor-associated antigen targeting with simultaneous delivery of stromal-disrupting payloads, enabling dual targeting of cancer cells and their supportive niche.

Switchable CAR platforms offer titratable control of CAR-T activity through modulating agents, enabling dynamic regulation of therapeutic intensity and potentially improving safety profiles by providing mechanisms to attenuate excessive immune activation when necessary [134].

Integration of synthetic biology principles has developed logic-gated CAR systems that sense and respond to multiple environmental inputs. These sophisticated circuits activate only in the presence of specific antigen combinations while remaining inactive in normal tissues expressing single antigens, enhancing specificity and reducing off-tumor toxicity [135].

### Personalization strategies and predictive biomarkers

Ovarian cancer heterogeneity necessitates personalized approaches to CAR-T therapy. Comprehensive tumor and microenvironment profiling, including multi-omic analysis, provides critical information for tailoring therapeutic strategies to individual patients. Integration of genomic, transcriptomic, and proteomic data identifies optimal target antigens, predicts potential resistance mechanisms, and guides selection of appropriate combination strategies [136].

Emerging biomarkers predictive of CAR-T response include factors related to target antigen expression, TME composition, and host immune status. Spatial transcriptomic analyses reveal that distribution and density of immunosuppressive elements significantly influence CAR-T infiltration and function. Quantitative imaging biomarkers from novel PET tracers show promise for non-invasive monitoring of CAR-T trafficking and persistence [47].

Patient-specific factors influencing T cell fitness represent critical determinants of therapeutic success [137]. Standardized functional assays assessing intrinsic T cell quality before manufacturing may identify patients requiring additional optimization strategies. Pharmacogenomic approaches examining genetic determinants of CAR-T efficacy and toxicity identify polymorphisms associated with differential outcomes, enabling further personalization [138].

### Translational challenges and proposed solutions

Manufacturing challenges, including generating sufficient CAR-T cells from heavily pre-treated patients with compromised T cell function, remain problematic. This is particularly relevant in ovarian cancer patients who typically receive multiple lines of lymphodepleting chemotherapy regimens that adversely affect T cell quantity, phenotype, and functionality [139]. Optimized manufacturing protocols incorporating selective expansion of specific T cell subsets with enhanced stemness properties, supplementation with homeostatic cytokines, and metabolic modulation may address these limitations. Allogeneic “off-the-shelf” CAR-T products offer potential solutions to manufacturing constraints but introduce complexities related to graft-versus-host disease and rejection.

Regulatory considerations for combination strategies present additional complexities. Developing rational, evidence-based combination approaches requires careful consideration of sequence, timing, and dosing to maximize synergistic effects while minimizing overlapping toxicities. Novel clinical trial designs, such as adaptive platform trials, may accelerate evaluation of multiple combination strategies.

Cost and accessibility barriers limit widespread implementation of CAR-T therapies. Current manufacturing processes remain labor-intensive and expensive. Development of automated, closed-system manufacturing platforms and point-of-care production systems may reduce costs and expand access. Regulatory initiatives targeting financial compensation frameworks and novel economic arrangements are essential to facilitate universal availability of these prospectively revolutionary therapeutic modalities [140].

### Integrated approaches for clinical implementation

Successful clinical implementation of CAR-T therapy for ovarian cancer requires integration into multimodal treatment regimens. Strategic sequencing with conventional therapies may create favorable conditions for CAR-T efficacy. Neoadjuvant applications of CAR-T therapy, prior to surgical intervention, leverage intact vasculature of primary tumors to facilitate CAR-T trafficking while generating systemic immune responses capable of addressing micrometastatic disease [141, 142].

Standardized assessment criteria specifically tailored to immunotherapeutic approaches in solid tumors are essential for accurate evaluation of clinical outcomes. Conventional response criteria may underestimate therapeutic benefit due to pseudoprogression and delayed response kinetics [143]. Integration of novel imaging modalities and molecular response assessments provides more comprehensive evaluation of therapeutic efficacy.

The evolution of CAR-T cell therapy for ovarian cancer stands at a pivotal juncture, with unprecedented opportunities to overcome historical limitations. Realization of this potential requires coordinated efforts across scientific disciplines, careful attention to translational considerations, and innovative approaches to clinical implementation. Integration of personalized strategies guided by comprehensive biomarker analysis, combined with continued refinement of CAR designs and manufacturing processes, offers a promising path forward for patients with ovarian cancer, for whom new therapeutic options are urgently needed.

## Conclusion

The tumor microenvironment (TME) in ovarian cancer presents formidable barriers to CAR-T cell therapy through a network of immunosuppressive mechanisms, including inhibitory cellular populations, checkpoint pathways, metabolic restrictions, and physical obstacles that collectively impair T cell trafficking, persistence, and cytotoxic function. Our analysis reveals that effective CAR-T strategies must address these multifaceted challenges through an integrated approach combining genetically engineered CAR-T cells resistant to TME-mediated suppression, rationally designed combination therapies targeting key immunosuppressive pathways, and innovative delivery systems enhancing tumor infiltration. While significant translational challenges remain, personalized implementation of these strategies guided by comprehensive TME profiling offers unprecedented potential to transform treatment outcomes for ovarian cancer patients, representing a critical advancement in gynecologic oncology. Collectively, current evidence indicates that immunosuppressive cellular components and inhibitory cytokine networks within the TME significantly impair CAR-T cell cytotoxicity, while dysregulated signaling pathways contribute to reduced infiltration and limited persistence of engineered T cells. In the near term, combinatorial strategies and improved CAR designs are expected to accelerate clinical translation, with ongoing efforts focusing on enhancing persistence, specificity, and safety. Future success may depend not solely on technological innovations but on integrative design frameworks that dynamically couple CAR-T function with patient-specific TME architecture and evolution.

## Data Availability

No datasets were generated or analyzed during the current study.
